# Spinning Top Urethra and Lower Urinary Tract Dysfunction in a Young Female

**DOI:** 10.1100/tsw.2004.55

**Published:** 2004-06-07

**Authors:** P.N. Dogra, M.S. Ansari

**Affiliations:** Department of Urology, All India Institute of Medical Sciences, New Delhi, India

## Abstract

Spinning top urethra (STU) denotes a particular urethral configuration that is a dilated posterior urethra mainly seen in young girls or women. STU deformity arises secondary to detrusor instability, leading to a rise the intravesical pressure against a closed sphincter. We describe a case of spinning top urethra in a 30-year-old woman who presented with lower urinary tract symptoms and left flank pain.

## INTRODUCTION

Spinning top urethra (STU) is a term used to describe a dilated posterior urethra, mainly seen in young girls or women. It has long been regarded as a normal variant in female and only urodynamic evaluation that raised the possibility that this variant is associated with bladder instability. We describe a case of spinning top urethra in a 30-year-old woman who present with lower urinary tract symptoms and left flank pain.

## CASE REPORT

A 30-year-old woman presented with lower urinary tract symptoms, consisting mainly of frequency and urgency for 10 years, and left flank pain for the last year. Routine laboratory investigations were within normal limits. A KUB showed left renal calculi. Intravenous urography (IVU) showed bilateral normal functioning kidneys with hydroureteronephrosis (Fig. 1). Uroflowmetry showed a normal flow rate. Voiding cystourethrogram (VCUG) showed a dilated posterior urethra with a constriction ring in the distal urethra (spinning top shape) (Fig. 2). Pressure flow study revealed detrusor instability with intermittent hyperactivity of the external sphincter at the time of unstable contraction during the filling phase. Cystoscopy did not reveal any obstruction in the urethra due to stricture. Percutaneous nephrolithotomy was performed to treat the left renal calculi. Presently, the patient is doing well on anticholinergics and intermittent self-catherization.

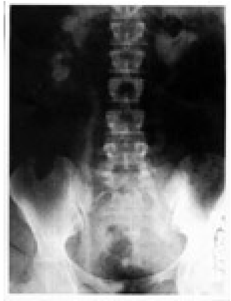

**FIGURE 1.** Intravenous urography (IVU) showing bilateral normal functioning kidneys with hydroureteronephrosis.

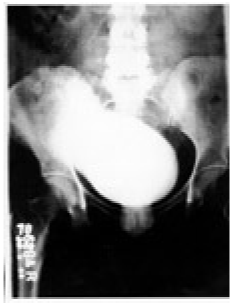

**FIGURE 2**. Voiding cystourethrogram (VCUG) showing the dilated posterior urethra with a constriction ring in the distal urethra (spinning top shape).

## DISCUSSION

Spinning top urethra (STU) simply denotes a dilated posterior urethra that has been described as a rare deformity in young women in association with lower urinary tract dysfunction. The pathophysiological mechanism proposed for this extreme disproportion in the urethra is bladder instability with concomitant voluntary sphincter contraction, leading to ballooning of the posterior urethra. This dilation will be maximal in subjects with weak bladder neck tone, as in congenital wide bladder neck anomaly (1). A male variant of STU is also described secondary to urethral valves, creating an obstructive kink in the urethra analogous to ureteropelvic junction obstruction (2). The condition needs to be distinguished from Lyon¹s ring, which is due to an organic obstruction secondary to a constrictive ring of fibrous tissue situated in the distal urethra of young girls (3).

VCUG in more than 50 percent of these patients is diagnostic or suggestive of detrusor instability with STU. The ideal study is still performed with video urodynamics, which show detrusor instability and the dilation of the posterior urethra in real time. Cystoscopy should be performed to rule out obstruction due to stricture. Encouragingly, treatment with anticholinergics, tricyclic antidepressants and bladder training are useful in more than 90 percent of patients. Intermittent self-catheterization may help prevent infection and deteriotration of the upper tracts. Some investigators have used urethral dilation with the Otis urethrotome, and reported excellent results both subjectively and urodynamically without urinary incontinence afterward (4). Diazepam and baclofen may be useful in some cases for relaxation of the voluntary sphincter spasms.

Finally, we stress the clinical importance of the pressure flow study with sphincter electromyography (EMG) and preferably fluoroscopic monitoring of the bladder and urethra when one encounters female patients with long histories of lower urinary tract symptoms to come to a more definitive diagnosis of such rare anomalies.
